# Long noncoding RNAs as versatile molecular regulators of cellular stress response and homeostasis

**DOI:** 10.1007/s00439-023-02604-7

**Published:** 2023-10-02

**Authors:** Julia Scholda, Thi Thuy Anh Nguyen, Florian Kopp

**Affiliations:** Faculty of Life Sciences, Department of Pharmaceutical Sciences, Clinical Pharmacy Group, University of Vienna, Josef-Holaubek-Platz 2, 1090 Vienna, Austria

## Abstract

Normal cell and body functions need to be maintained and protected against endogenous and exogenous stress conditions. Different cellular stress response pathways have evolved that are utilized by mammalian cells to recognize, process and overcome numerous stress stimuli in order to maintain homeostasis and to prevent pathophysiological processes. Although these stress response pathways appear to be quite different on a molecular level, they all have in common that they integrate various stress inputs, translate them into an appropriate stress response and eventually resolve the stress by either restoring homeostasis or inducing cell death. It has become increasingly appreciated that non-protein-coding RNA species, such as long noncoding RNAs (lncRNAs), can play critical roles in the mammalian stress response. However, the precise molecular functions and underlying modes of action for many of the stress-related lncRNAs remain poorly understood. In this review, we aim to provide a framework for the categorization of mammalian lncRNAs in stress response and homeostasis based on their experimentally validated modes of action. We describe the molecular functions and physiological roles of selected lncRNAs and develop a concept of how lncRNAs can contribute as versatile players in mammalian stress response and homeostasis. These concepts may be used as a starting point for the identification of novel lncRNAs and lncRNA functions not only in the context of stress, but also in normal physiology and disease.

## Introduction

Each organism and each cell have to maintain homeostasis to ensure their biological integrity. Homeostasis is constantly challenged by endogenous and exogenous stress conditions, such as hypoxia, DNA damage, oxidative stress, heat shock, nutrient deprivation or viral infections. To overcome these harmful conditions and to maintain homeostasis and ensure healthy aging, every cell must activate a range of conserved stress response pathways to prevent pathophysiological consequences (Kourtis and Tavernarakis 2011). While the response to cellular stress can be either specific for or shared between different stress and cell types, all stress response pathways have in common that they enable the cell to integrate different inputs of cellular stress, adapt to them and finally overcome them by either restoring homeostasis or inducing cell death (Fulda et al. 2010). Several key stress proteins and pathways have been identified that can integrate various stress signals and induce a multifaceted stress response. Among those regulators are for example the tumor suppressor protein p53, the mechanistic target of rapamycin (mTOR) or the eukaryotic initiation factor 2 (eIF2) protein kinases of the integrated stress response. p53 is activated upon various types of stress, such as DNA damage, hypoxia or oncogene activation, and its activation leads to the induction of the *cyclin-dependent kinase inhibitor 1A* (*CDKN1A* encoding p21) and other key response genes that regulate cell cycle arrest, apoptosis and senescence (Levine et al. 2006). mTOR is critical for cell growth and proliferation under nutrient- and growth factor-rich conditions and is turned off upon metabolic stress, nutrient starvation, hypoxia and DNA damage (Aramburu et al. 2014). The integrated stress response, on the other hand, is induced by a broad range of cellular stress conditions, including amino acid deprivation, viral infections and endoplasmic reticulum (ER) stress. It results in phosphorylation of eIF2α and consequently in global reduction of protein translation and preferential translation of stress-related proteins, which initiates an adaptive program to overcome the stress (Pakos-Zebrucka et al. 2016). In addition to these molecularly defined stress response pathways, the cell can also employ a less defined response that relies on the formation of dynamic cellular condensates that emerge from multivalent RNA and protein interactions. Such ribonucleoprotein condensates, also referred to as stress granules, have been shown to play an integral role in response to several different types of stress and in the control of normal physiology and disease (Jeon et al. 2022; Lee and Namkoong 2022). Independent of the underlying molecular mechanism of the different cellular stress response pathways, they all need to be tightly regulated with the ability to quickly adapt to stress, to integrate multiple stress signals and to fine-tune the response to certain cellular stress conditions in order to ensure an adequate output of the stress response.

The human genome is extensively transcribed into several types of non-protein-coding RNA species, some of which have been shown to act as potent regulators and mediators of the cellular stress response in animals as well as in plants (Jha et al. 2020; Mendell and Olson 2012). A relatively novel class among these noncoding RNA species represent the long noncoding RNAs (lncRNAs), which are defined by a sequence length of more than 500 nucleotides (Mattick et al. 2023). With regard to protein-coding genes, lncRNAs can be produced as intergenic, antisense, intronic or overlapping transcripts. They lack a detectable open reading frame, are mostly transcribed by RNA polymerase II, often spliced and polyadenylated, and may also occur in a circular form. Therefore, lncRNAs comprise a heterogeneous class of noncoding RNAs that have diverse physiological and molecular functions (Ulitsky and Bartel 2013). While tens of thousands of lncRNA genes have been annotated (Volders et al. 2015), the biological and molecular characterization of the vast majority of them is still missing. This is, at least in part, due to the fact that each lncRNA candidate gene has to be studied on a case-by-case basis to infer a meaningful biological and molecular function for the encoded transcript. Hence, there is only a very limited number of lncRNAs that have well-established biological roles with solid experimental validation of their molecular modes of action (Rinn and Chang 2020). In principle, lncRNA functions can be categorized into two different modes of action: (I) regulation of gene expression and transcription of neighboring genes in *cis* and (II) regulation of nuclear and cytoplasmic processes in *trans*. Such functions in *trans* include for example the modulation of gene expression at distant genomic loci, the formation of nuclear and cytoplasmic structures and compartments, or the regulation of interacting protein and/or RNA molecules. Of note, some *trans*-acting lncRNA genes have been also shown to encode small peptides that can exert important biological functions (Makarewich and Olson 2017; Patop et al. 2019; Wright et al. 2022). Depending on the proposed molecular mode of action, a lncRNA has to fulfill certain important criteria. For instance, if a lncRNA is proposed to function in *trans* via the interaction with other biomolecules, such as proteins or other RNAs like miRNAs, the respective lncRNA often needs to be expressed at sufficient abundance with ideally conserved sequences or secondary structures that confer specificity for its interaction with other biomolecules (Kopp and Mendell 2018). On the other hand, if lncRNAs are proposed to function in *cis* to regulate transcription and gene expression of neighboring genes, it needs to be demonstrated whether the accumulation of the actual encoded lncRNA sequence is necessary for the observed biological effect or whether the sole act of transcription or even DNA elements within the lncRNA locus are sufficient to control the neighboring gene locus independent of the encoded lncRNA (Anderson et al. 2016; Engreitz et al. 2016).

Increasing evidence suggests that lncRNAs can function as versatile regulators of the mammalian stress response via diverse molecular mechanisms that facilitate a time- and dose-sensitive response to various cellular stress conditions. In this review, we describe novel insights into lncRNA function in the mammalian stress response and homeostasis. We highlight and elaborate on the different molecular modes of action of lncRNAs that have been experimentally validated to regulate, mediate or titrate the response to a range of cellular stress conditions. We aim to generate a framework for the functional classification of stress-related lncRNAs based on their molecular modes of action, which we divide into three general classes: (I) lncRNAs regulating gene expression in *cis* or *trans*, (II) lncRNAs acting as scaffolds or tethers in ribonucleoprotein complexes, and (III) lncRNAs regulating and forming cellular condensates.

## lncRNA-mediated regulation of gene expression upon cellular stress

Gene regulatory functions in the nucleus were among the first described roles for lncRNAs (Lee 2012). It has been suggested as a common mode of action that lncRNAs bind to certain RNA binding proteins or components of the polycomb repressor complex and guide these to either local (regulation in *cis*) or distant (regulation in *trans*) genomic sites, where they can modulate the chromatin state and repress or activate gene expression of the respective gene locus. Technological advances, including genome editing and rigorous biochemical testing of specific RNA-protein interactions, have led to the awareness that this initial model of how lncRNAs generally regulate gene expression in the nucleus may need to be revised (Kopp and Mendell 2018). The oftentimes low copy numbers of lncRNAs together with the usually promiscuous binding of RNA binding proteins to lncRNAs have challenged the proposed gene regulatory functions of some lncRNAs acting in *trans*, whereas careful studies of lncRNA loci using precise genetic models to distinguish between the role of transcription, splicing and the actual lncRNA transcript in lncRNA-mediated regulation of local gene expression have questioned the proposed modes of action for some lncRNAs acting in *cis*. Although these originally predicted gene regulatory functions of lncRNAs in *cis* and *trans* may not be as common as initially anticipated, they still represent an important mode of action that is also utilized and critical for lncRNA-mediated stress response.

**Fig. 1:**
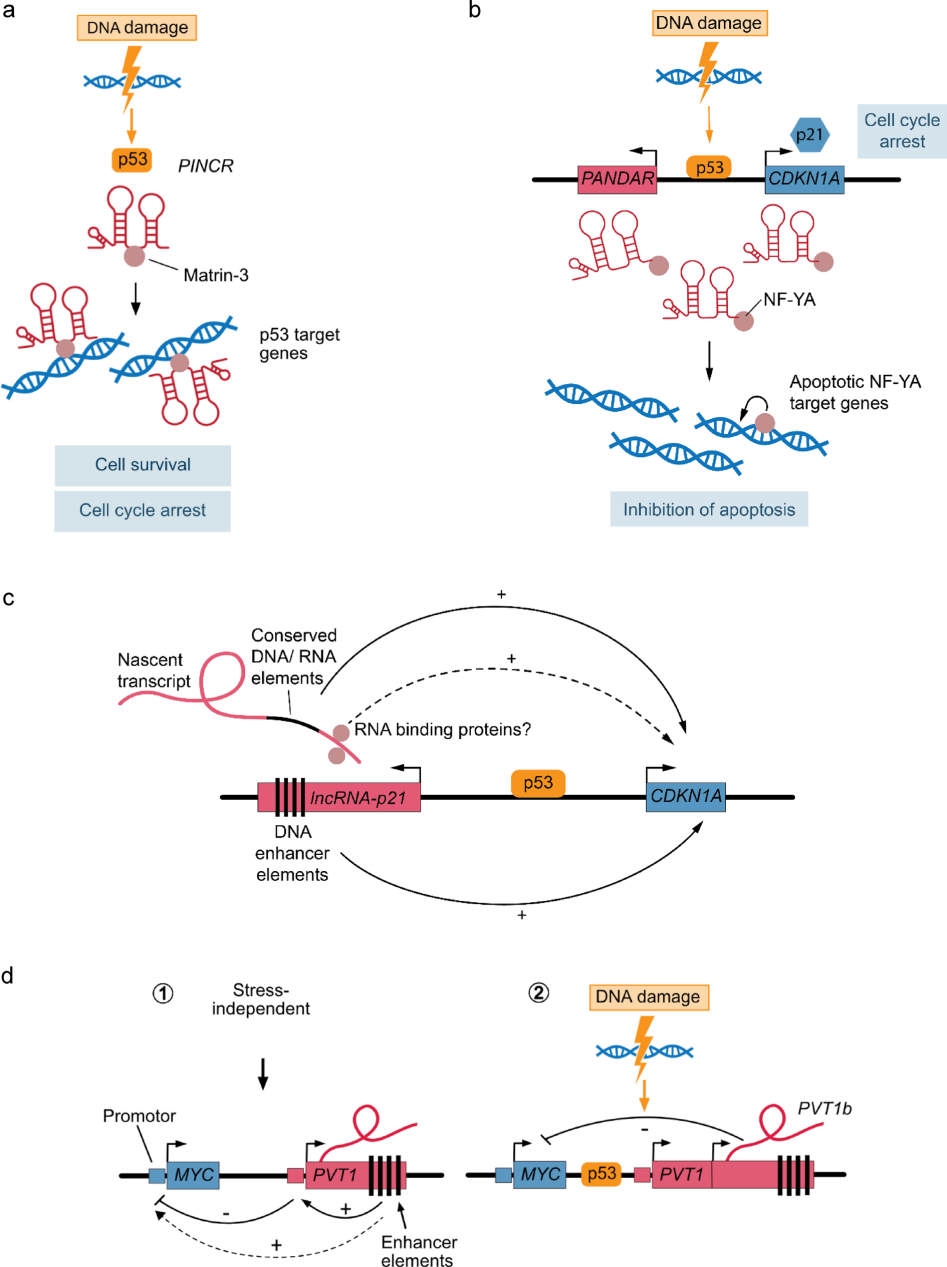
lncRNA-mediated regulation of gene expression upon cellular stress. **a** *PINCR*-mediated recruitment of matrin-3 to selected p53 target genes upon DNA damage and subsequent regulation of their transcription in *trans*. **b** Induction of *PANDAR* expression from the *CDKN1A* locus upon DNA damage and regulation of NF-YA and its target genes in *trans*. **c** Local gene regulation of *CDKN1A* by either nascent transcription through conserved DNA/RNA elements or activating DNA enhancer elements in the *lincRNA-p21* locus. **d** Promoter competition between the *MYC* and *PVT1* locus (1) and RNA- or transcription-dependent regulation of *MYC* by *PVT1b* (2)

Several noncoding RNAs, including lncRNAs, are regulated by the tumor suppressor protein p53 (Chaudhary and Lal 2017). The p53 pathway is typically activated upon DNA damage, which results in pleiotropic cellular effects, ranging from cell cycle arrest to pro-survival and pro-apoptotic signals. One such lncRNA represents the *p53-induced noncoding RNA* (*PINCR*), a ~ 2.2 kilobase (kb) long intergenic lncRNA that is predominantly localized to the nucleus, expressed from the X-chromosome and highly induced upon doxorubicin treatment in a p53-dependent manner (Chaudhary et al. 2017). *PINCR* has been shown to be critical for G1 cell cycle arrest and anti-apoptotic effects upon DNA damage in colorectal cancer cells, and *PINCR* loss-of-function results in increased sensitivity to doxorubicin and 5 fluorouracil (5-FU) treatment. On a molecular level, *PINCR* interacts with and recruits the nuclear protein matrin-3 through a yet unknown mechanism to a subset of p53 target genes. Matrin-3 then binds to p53 in a DNA- and RNA-independent fashion and forms regulatory chromatin loops with surrounding enhancer elements to regulate a subset of p53 target genes (Fig. 1a). *PINCR* therefore represents a stress-induced lncRNA that can modulate the p53-mediated response to DNA damage in *trans* via the regulation of selected p53 target genes that are important for G1 cell cycle arrest and cell survival. In a screen for RNAs transcribed from cell cycle-related gene promoters, several new putative lncRNAs have been identified, five of which are induced by DNA damage and arise from the p53-regulated *CDKN1A* (p21) promoter region (Hung et al. 2011). Among those lncRNAs is a ~ 1.5 kb long noncoding transcript termed *p21 associated noncoding RNA DNA damage activated* (*PANDA*) or *promoter of CDKN1A antisense DNA damage activated RNA* (*PANDAR*) that is located ~ 5 kb upstream of the *CDKN1A* transcription start site and highly induced upon DNA damage in a p53-dependent manner. Silencing of *PANDAR* does not affect the expression of its neighboring gene *CDKN1A* and results in increased sensitivity of human fibroblasts to doxorubicin treatment. *PANDAR* binds to and sequesters the transcription factor nuclear transcription factor Y subunit alpha (NF-YA), thereby limiting the expression of pro-apoptotic NF-YA target genes (Fig. 1b). Although the specificity of the interaction between *PANDAR* and NF-YA is not fully clear, it has been shown that siRNA-mediated knockdown of NF-YA is able to rescue and reduce apoptosis in *PANDAR*-depleted cells, demonstrating a critical role of NF-YA downstream of *PANDAR*. Taken together, these findings give an example of a DNA damage-induced lncRNA from the *CDKN1A* locus that acts in *trans* as a molecular decoy for the transcription factor NF-YA. *PANDAR* fine-tunes the DNA damage response of p53 via the regulation of pro-apoptotic NF-YA target genes, whereas other p53 effectors, such as p21, may rather regulate other aspects like cell cycle arrest. Hence, *PINCR* as well as *PANDAR* are two examples of lncRNAs that are integrated into the p53-mediated DNA damage response and that regulate the targeted expression of selected p53 response genes in *trans* through two distinct mechanisms.

Another lncRNA that is transcribed upstream of the *Cdkn1a* locus and induced by DNA damage in a p53-dependent manner is the noncoding RNA *lincRNA-p21*. Several different modes of action have been proposed for *lincRNA-p21* since its discovery in 2010 (Huarte et al. 2010), ranging from gene regulatory functions in *trans* (Huarte et al. 2010) to post-transcriptional regulation of selected mRNA targets (Yoon et al. 2012) and gene regulation of its neighboring gene *Cdkn1a* (p21) in *cis* (Dimitrova et al. 2014; Groff et al. 2016; Winkler et al. 2022). Initially, *lincRNA-p21* was reported to globally repress genes in the p53 pathway necessary for proper apoptosis induction upon DNA damage (Huarte et al. 2010). It was proposed that, upon p53-mediated induction, *lincRNA-p21* binds to and recruits the RNA binding protein heterogeneous nuclear ribonucleoprotein K (hnRNPK) to selected p53 target genes, which are subsequently repressed. In a later study (Dimitrova et al. 2014), however, Dimitrova et al. showed that *lincRNA-p21* rather activates the transcription of *Cdkn1a* (p21) in *cis*, presumably via interaction with hnRNPK, which then leads to a global repression of polycomb repressor complex 2 (PRC2) target genes, affecting the G1/S cell cycle checkpoint and cell proliferation. Importantly, this was the first study demonstrating a *cis* regulatory function of *lincRNA-p21* using an allele-specific mouse model of *lincRNA-p21* and *Cdkn1a* deficiency. Later, it was found that *lincRNA-p21* deletion in mice results in global changes of local gene expression, including repression of the *Cdkn1a* gene, which is independent of the respective *lincRNA-p21* expression in the tested tissues (Groff et al. 2016). These effects were attributed to activating enhancer elements within the *lincRNA-p21* locus, which can form chromosomal loops with neighboring genes and induce their transcription, suggesting a role for *lincRNA-p21* as an enhancer RNA (eRNA) whose expression is not required for the gene regulatory function of this locus. In a recent study (Winkler et al. 2022), the model of *lincRNA-p21*’s mode of action was further refined. Through a series of elegant genetic models that each independently address the role of transcription termination, transcript degradation, splicing inhibition, deletion of conserved regions, deletion of the *lincRNA-p21*-resident p53 response element, and transcriptional interference, it could be shown that *lincRNA-p21* regulates *Cdkn1a* expression in *cis* through transcription of conserved DNA or RNA elements in the nascent lincRNA*-p21* transcript, while transcription and splicing as well as the accumulation of full-length *lincRNA-p21* are not required. Taken together, *lincRNA-p21* can fine-tune the response to cellular stress in the p53 pathway via the regulation of the neighboring gene *Cdkn1a*. Whether the nascent transcript recruits factors, such as hnRNPK, to the *Cdkn1a* locus or whether rather the transcription of the encoded conserved DNA elements confer the *cis* regulatory effect remains an open question. In some cell and tissue types, the sole existence of regulatory DNA enhancer elements in the *lincRNA-p21* locus may already suffice to regulate *Cdkn1a* in *cis* without the necessity of transcriptional activity (Fig. 1c).

*Plasmacytoma variant 1* (*Pvt1*) represents a lncRNA transcribed approximately 50 kb downstream of the *myeolocytomatosis* (*Myc*) oncogene that has been initially identified as a hotspot for chromosomal translocations in murine and later also in human lymphomas (Cory et al. 1985; Graham and Adams 1986; Graham et al. 1985). Since the human genomic locus on chromosome 8 (8q24), including the *PVT1* gene, has been shown to be amplified in human breast cancer (Curtis et al. 2012) and *PVT1* itself has been reported to stabilize the MYC protein (Tseng et al. 2014), *PVT1* has been described as a potential oncogene. However, this notion has become challenged by the finding that the *PVT1* promoter competes with the *MYC* promoter for enhancer elements within the *PVT1* locus, a function that appears to be independent of the *PVT1* transcript (Cho et al. 2018). This negative regulation of *MYC* expression together with the fact that mutations in the *PVT1* promoter frequently occur in human cancers suggest a rather tumor suppressive role for the *PVT1* locus and promoter in human cancers. Interestingly, there is also a p53 response element within the *PVT1* locus that results in p53-mediated repression of *MYC* upon genotoxic stress. *MYC* repression is thereby important for the general reduction in transcription and the proper induction of cell cycle arrest and apoptosis (Porter et al. 2017). Recently, it has been shown that oncogenic as well as genotoxic stress can specifically induce the expression of a certain p53-dependent *Pvt1* isoform, called *Pvt1b*, in mouse embryonic fibroblasts (Olivero et al. 2020). *Pvt1b* negatively regulates the expression of the neighboring *Myc* oncogene and inhibits tumor growth, but not progression, in a murine lung tumor model. Interestingly, the *Pvt1b* RNA appears to be critical for stress-induced repression of *Myc*. Enforced production of the *Pvt1b* transcript, even in the absence of cellular stress and functional p53, is sufficient to inhibit *Myc* expression in *cis*. Based on the existing literature, and besides reported oncogenic functions, the *PVT1* locus appears to act as a tumor suppressor through at least two distinct molecular mechanisms: one is p53- and RNA-independent via promoter competition between *PVT1* and *MYC* (Cho et al. 2018), and another one is activated upon cellular stress, which depends on p53 as well as on the RNA (specifically, the *Pvt1b* isoform) (Olivero et al. 2020) (Fig. 1d). The complex regulation of *MYC* by the *PVT1* locus illustrates that a lncRNA gene can modulate cellular programs in different cellular conditions, such as tumorigenesis and response to genotoxic stress, through various modes of action depending on the cellular context.

## lncRNAs as stress-induced scaffolds or tethers

In addition to directly regulating transcription and gene expression in *cis* or *trans*, lncRNAs can form ribonucleoprotein complexes in order to control the composition of higher order cellular complexes as well as the activity or stability of the interacting protein and/or RNA molecules. This role of lncRNAs, which can be described as a scaffolding function, has been shown to be also utilized by noncoding RNAs that are involved in the mammalian stress response.

**Fig. 2:**
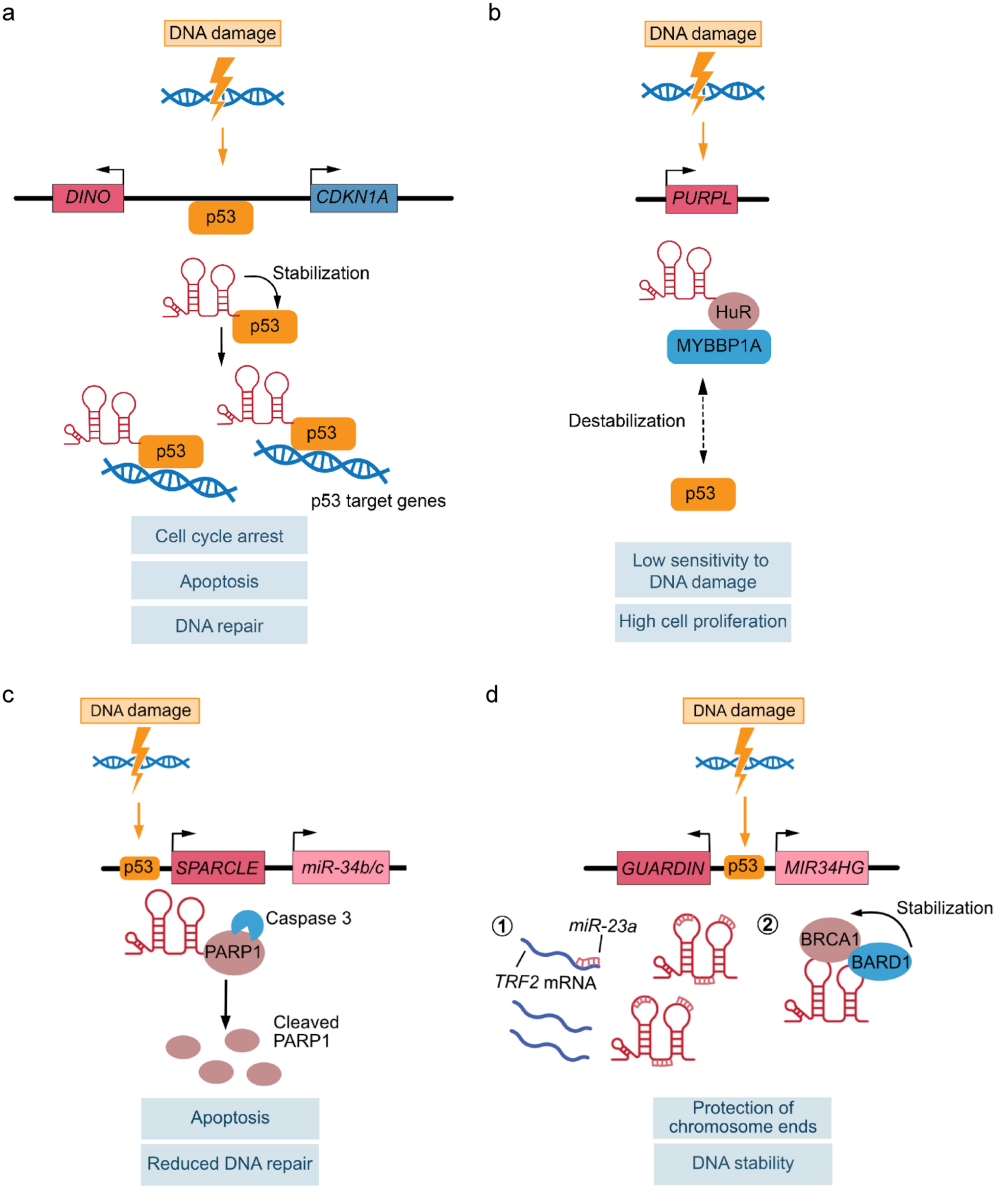
lncRNAs as stress-induced scaffolds or tethers (I). **a** *DINO*-mediated stabilization of the p53 protein upon DNA damage. **b** Maintenance of basal p53 levels through p53 destabilization by the lncRNA *PURPL*. **c** *SPARCLE*-catalyzed cleavage of PARP1 by caspase 3 upon DNA damage. **d** Dual mode of action of the lncRNA *GUARDIN* upon DNA damage, (1) as a competing endogenous RNA for *miR-23a*, and (2) as a scaffold in a ribonucleoprotein complex stabilizing BRCA1

In a screen for transcripts that originate from promoters of human cell cycle genes (Hung et al. 2011), a novel ~ 1 kb long lncRNA has been identified as a divergent transcript originating from the *CDKN1A* promoter. This lncRNA has been shown to be highly induced by DNA damage in a p53-dependent manner and has been named *damage induced noncoding* or *DINO* (Schmitt et al. 2016). *DINO* induced by DNA damage or by ectopic overexpression binds to the C-terminal region of p53 via a distinct p53 binding site, resulting in the stabilization of the p53 protein. It is not yet fully understood how *DINO* binding stabilizes p53 protein and whether there are other proteins involved in the *DINO*-p53 ribonucleoprotein complex. However, it has been demonstrated that this positive feed forward loop and p53 stabilization are required for robust p53 target gene expression and proper induction of cell cycle arrest and apoptosis upon sustained DNA damage. *Dino* knockout mice display similarly reduced p53 levels as p53 heterozygous knockout mice and a comparable decreased survival upon lethal doses of irradiation, highlighting the importance of appropriate p53 protein levels under steady state and stress conditions. Interestingly, later studies (Marney et al. 2021, 2022) revealed that the *Dino*/*Cdkn1a* locus represents a tumor suppressor locus as loss of *Dino* can promote lymphoma in an *Eµ-Myc* mouse model as well as induce the formation of a subset of spontaneous tumors, including sarcomas and lymphomas. In line with *Dino*’s role in the p53 pathway, *Dino* requires intact p53 for its tumor suppressor function, while loss or inactivation of p53 abrogates its tumor suppressive role in lymphoma (Marney et al. 2021). Consistently, *DINO* promoter hypermethylation can be often found in human cancers in a mutually exclusive manner with p53 mutations, potentially explaining the escape from tumor suppression in human cancers with intact p53. These findings suggest that *DINO* forms a positive feed forward loop in the p53 pathway to titrate the response to DNA damage via the direct interaction with and stabilization of p53, leading to an amplification of the output of the DNA damage response once it exceeds a certain threshold (Fig. 2a). Another lncRNA that is induced upon DNA damage in a p53-dependent manner and that regulates p53 stability is the *p53 upregulated regulator of p53 levels* or *PURPL* (Li et al. 2017). In contrast to the afore-discussed lncRNA *DINO* that stabilizes p53, *PURPL* has been shown to suppress p53 protein stability and basal expression levels. *PURPL* associates with the p53 stabilizing protein MYBBP1A (MYB binding protein 1a) via the RNA binding protein HuR, preventing the formation of the MYBBP1A-p53 complex and hence the stabilization of p53. Accordingly, *PURPL* loss-of-function results in elevated basal p53 protein levels in colorectal cancer cells and consequently in reduced cell and tumor growth as well as in increased sensitivity to DNA damage. Of note, silencing of MYBBP1A in *PURPL* knockout cells partially restores basal p53 protein levels and normal cell proliferation, confirming an important role of MYBBP1A downstream of *PURPL* (Fig. 2b). These results suggest that *PURPL* is part of an autoregulatory, negative feedback loop that keeps basal p53 levels in check and facilitates proper cell proliferation in colorectal cancer cells. Overall, the modes of action of these two lncRNAs, *DINO* and *PURPL*, are intriguing examples of how lncRNAs can directly and timely influence the pathway they are integrated in, which can have important implications in human health and disease, including tumorigenesis.

Besides the direct regulation of p53, lncRNAs can also act as downstream effectors of p53 in the DNA damage response. The lncRNA *SPARCLE* is a ~ 770 nt long, nuclear RNA that is located upstream of the *microRNA-34b/c* (*miR-34b/c*) cluster on chromosome 11 (Meza-Sosa et al. 2022). Upon DNA damage, *SPARCLE* is induced in a p53-dependent manner together with *miR-34b/c* using the same p53 response element just upstream of the *SPARCLE* transcription start site. Interestingly, *SPARCLE* lacks polyadenylation and may be processed from the *miR-34b/c* precursor. Its expression level, even after induction of DNA damage, is low at around 10 copies per cell on average. Nevertheless, loss of *SPARCLE* enhances DNA repair and inhibits DNA damage-induced apoptosis, which is comparable to p53 loss-of-function and can be rescued by *SPARCLE* overexpression. While *SPARCLE* does not appear to regulate transcription, it binds with high affinity to poly(ADP-ribose) polymerase 1 (PARP1) and serves as a cofactor for caspase 3. In the presence of *SPARCLE*, caspase 3 efficiently cleaves PARP1, which results in impaired DNA repair and increased apoptosis. Of note, *SPARCLE* can potently enhance caspase 3-mediated cleavage of PARP1 at very low molar ratios compared to PARP1, corroborating the proposed molecular mechanism even at low copy numbers. In line with these findings, expression of cleaved N-terminal PARP1 in *SPARCLE*-deficient cells results in reduced DNA repair and enhanced apoptosis upon DNA damage, suggesting that *SPARCLE* mainly acts by promoting caspase 3-mediated cleavage of PARP1. As *SPARCLE* is not induced before day one after DNA damage, it may be responsible to ensure cell death of cells with extensive DNA damage at later stages of the DNA damage response. *SPARCLE* thereby acts as a scaffold that facilitates PARP1 cleavage by caspase 3, regulating DNA repair and apoptosis (Fig. 2c). Through its high affinity to PARP1 in the nanomolar range and its presumably reversible binding to full length as well as cleaved PARP1, *SPARCLE* may function as a catalytic lncRNA that can perform its molecular mode of action even at the observed low copy numbers.

A novel lncRNA termed *GUARDIN* has been recently characterized as another downstream effector of p53 and described to be induced upon genotoxic stress and oncogene activation in a p53-dependent manner (Hu et al. 2018). *GUARDIN* is expressed as a divergent transcript from the promoter of the *miR-34 host gene* (*MIR34HG*), sharing the same promoter and p53 binding region. Importantly, interfering with *GUARDIN* levels does neither affect the expression of the neighboring and partly overlapping *MIR34HG* nor the encoded *miR-34*, ruling out a gene regulatory function in *cis*. *GUARDIN* displays nuclear as well as cytoplasmic subcellular localization and shows higher expression levels in human colon tumors with wild-type than mutant p53. Inhibition of *GUARDIN* in different human cancer cells impairs cancer cell proliferation and survival as well as tumor growth in vivo. Furthermore, *GUARDIN* inhibition promotes chromosome end-to-end fusion and DNA damage, resulting in increased apoptosis and senescence as well as augmented sensitivity to genotoxic stress (Hu et al. 2018). On a molecular level, a dual mode of action was proposed for *GUARDIN* function (Fig. 2d): (I) *GUARDIN* can act as a competing endogenous RNA (ceRNA) regulating *miR-23a* and its downstream mRNA target *telomeric repeat-binding factor 2* (*TRF2*), a critical component of the shelterin protein complex that is necessary for the protection of chromosome ends; (II) *GUARDIN* interacts with the proteins BRCA1 (breast cancer gene 1) and BARD1 (BRCA1 associated RING domain 1) via distinct binding regions, forming a ternary complex that stabilizes BRCA1, a key protein in the repair of DNA double strand breaks, by preventing its ubiquitination and proteasome-mediated degradation. Of note, the stoichiometry in terms of copy numbers and binding sites is plausible for the proposed modes of action as a ceRNA for *miR-23a* as well as a scaffold for the formation of the BRCA1-BARD1 complex. Additional evidence for a dual mode of action is provided by the fact that both TRF2 and BRCA1 overexpression is required to restore the cytoprotective effect of *GUARDIN* in *GUARDIN*-depleted cells. Taken together, this lncRNA is an example of a noncoding RNA that can exert pleiotropic functions in a ribonucleoprotein complex to maintain DNA integrity not only after genotoxic stress, but also at steady state under homeostatic conditions to preserve chromosomal stability.

**Fig. 3:**
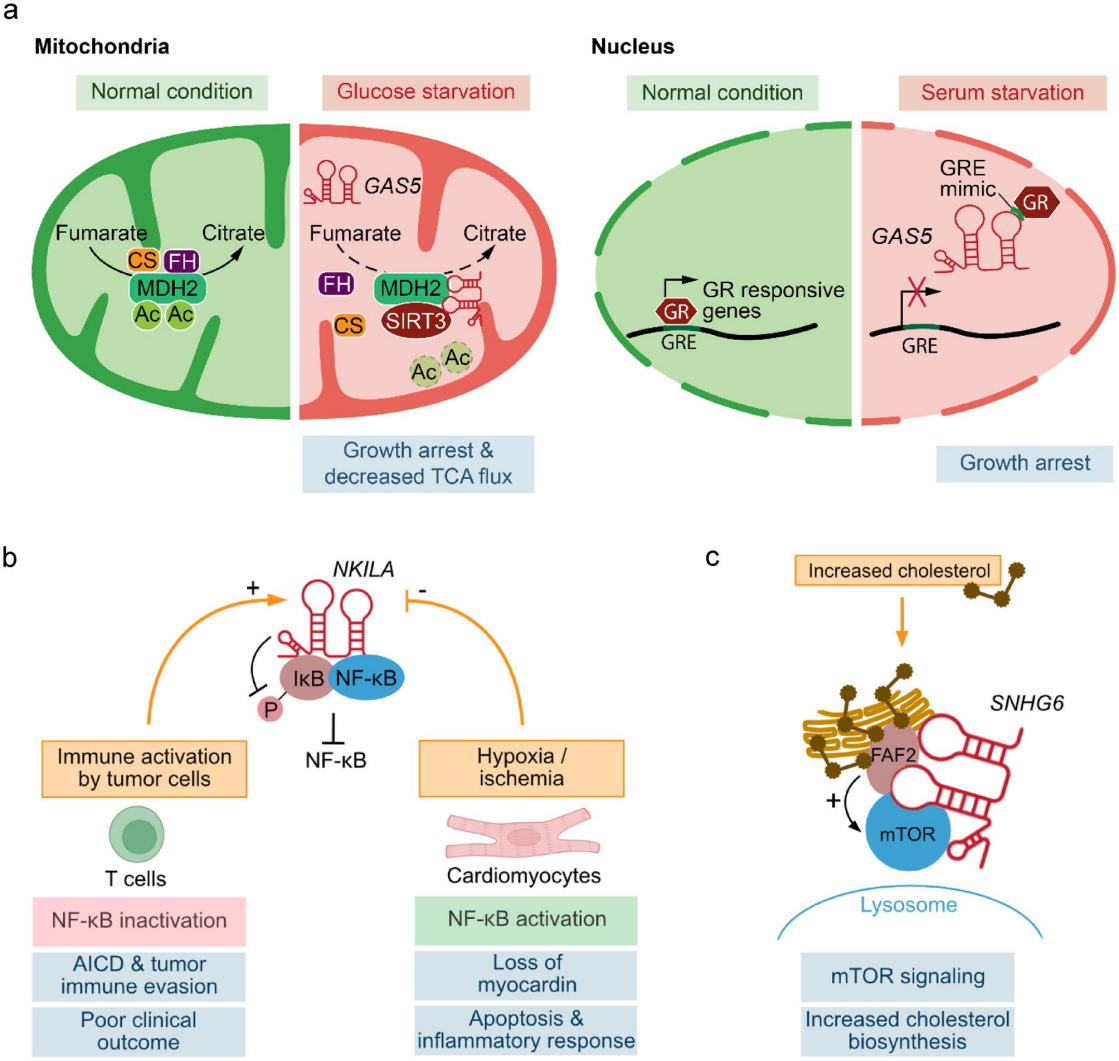
lncRNAs as stress-induced scaffolds or tethers (II). **a** Induction of *GAS5* upon glucose and serum starvation and subsequent regulation of mitochondrial and nuclear functions. (FH = fumarate hydratase, MDH2 = malate dehydrogenase 2, CS = citrate synthase, Ac = acetylation, TCA = tricarboxylic acid, GR = glucocorticoid receptor, GRE = glucocorticoid response elements). **b** Regulation of the NF-κB pathway by the lncRNA *NKILA* in different cellular stress conditions. (AICD = activation-induced cell death). **c** Activation of mTOR and cholesterol biosynthesis by the cholesterol-induced lncRNA *SNHG6*

Another example of a lncRNA that has been associated with cellular stress response and that has been ascribed more than one molecular mode of action is the lncRNA *growth arrest specific 5* (*GAS5*). *GAS5* was initially identified in a cDNA library of RNAs enriched in growth-arrested cells (Schneider et al. 1988). It is a highly abundant, spliced and polyadenylated lncRNA that hosts several small nucleolar RNAs (snoRNAs) in its introns (Coccia et al. 1992; Smith and Steitz 1998). Nutrient starvation often leads to growth arrest and eventually to cell death. Cell growth and survival depend on nutrients and can be modulated by glucocorticoid receptors upon starvation. Upon serum withdrawal, *GAS5* is strongly induced and binds to the DNA binding domain of the glucocorticoid receptor via a conserved glucocorticoid response mimic, modulating glucocorticoid receptor-regulated genes as well as cell survival and metabolic activity (Hudson et al. 2014; Kino et al. 2010). A structural analysis of the *GAS5* RNA using selective 2’ hydroxyl acylation analyzed by primer extension by mutational probing (SHAPE-MaP) revealed a modular structure of this noncoding RNA that consists of three modules, indicating that this lncRNA may serve pleiotropic functions (Frank et al. 2020): (I) a 5’ module that inhibits cell growth independent of steroid receptors, (II) a steroid receptor module that inhibits steroid-dependent cell growth, and (III) a structured core module that regulates mTOR-mediated inhibition of cell growth. In a recent study (Sang et al. 2021), an additional mode of action for *GAS5* was identified. In a screen for subcellular localization and organelle-enrichment of lncRNAs, it was shown that *GAS5* is localized to the mitochondrial fraction and that this mitochondrial localization is even more enriched upon glucose starvation. *GAS5* interacts with MDH2 and negatively regulates the formation of the FH-MDH2-CS (fumarate hydratase-malate dehydrogenase 2-citrate synthase) complex, which catalyzes the reaction of fumarate to citrate in the tricarboxylic acid (TCA) cycle, via sirtuin 3 (SIRT3)-mediated deacetylation of MDH2. Nutrient deprivation leads to *GAS5*-mediated reduction of the TCA flux and hence to growth arrest. These findings demonstrate once more that a lncRNA can have more than one mode of action and that these different molecular functions can be defined by the modular architecture of the lncRNA. In case of *GAS5*, the lncRNA can function in different cellular compartments either as a molecular decoy for glucocorticoid receptors or as a scaffold that modulates the activity of an enzyme complex of the TCA cycle, depending on the respective cellular demands and stress condition (Fig. 3a).

The immune-responsive NF-κB interacting lncRNA *NKILA* is another example of a lncRNA that can exert various physiological functions depending on the cellular and stress-related context. *NKILA* has been initially identified as a cytoplasmic lncRNA that is induced by inflammatory cytokines via the NF-κB pathway in human breast cancer cells (Liu et al. 2015). It binds to the transcription factor NF-κB and forms a stable ternary complex together with IκB, which prevents IκB phosphorylation and NF-κB activation. While decreased *NKILA* expression levels in breast tumors are associated with metastasis and poor prognosis of breast cancer patients (Liu et al. 2015), *NKILA* has been more recently shown to be also critical for immune evasion of breast and lung cancer cells (Huang et al. 2018). Tumor-specific cytotoxic T lymphocytes can detect and eliminate tumor cells at early stages. This tumor immunosurveillance, however, can be impaired by activation-induced cell death (AICD) of T lymphocytes, a process that removes activated T lymphocytes and contributes to tumor immune evasion. *NKILA* is induced in activated T cells by STAT1 (signal transducer and activator of transcription 1) in a calcium/calmodulin-dependent fashion and promotes AICD in tumor-specific cytotoxic T lymphocytes and T helper cells type 1 (T_H_1) via the inhibition of the NF-κB pathway. Interestingly, tumor-specific cytotoxic T cells and T_H_1 isolated from breast and lung cancer patients display a high expression of *NKILA* and a high sensitivity to AICD. Conversely, silencing *NKILA* in the context of an adoptive cell therapy in a patient-derived xenograft model of breast cancer can reduce AICD in T lymphocytes and improve therapy efficacy by preventing tumor immune evasion (Huang et al. 2018). Moreover, *NKILA* has been shown to be downregulated upon ischemia–reperfusion injury in cardiomyocytes, which results in the activation of the NF-κB pathway and a loss of myocardin, leading to increased apoptosis and inflammatory responses (Liu et al. 2020). Overexpression of *NKILA*, on the other hand, can improve myocardial ischemic injury by inhibiting NF-κB signaling and restoring myocardin levels. These findings demonstrate how a lncRNA can regulate different physiological outcomes depending on the respective cell and stress type via a single molecular mode of action, which is to function as a scaffold that binds NF-κB and IκB, thereby preventing IκB phosphorylation and NF-κB activation (Fig. 3b).

Another lncRNA that has been recently reported to function as a molecular scaffold is the *small nucleolar RNA host gene 6* (*SNHG6*) (Liu et al. 2022). *SNHG6* is significantly upregulated in hepatoma compared to normal liver tissue and has been shown to be a cholesterol effector that accelerates progression from non-alcoholic fatty liver disease (NAFLD) to hepatocellular carcinoma (HCC). Upon stimulation with cholesterol, *SNHG6* expression is induced and associated with the endoplasmic reticulum (ER) and lysosomal compartments. *SNHG6* forms a complex with the ER-associated protein Fas-associated factor family member 2 (FAF2) and mTOR at the ER-lysosome contact sites. This formation is regulated by cholesterol levels and requires *SNHG6* for mTOR recruitment to the lysosomes and FAF2-mediated activation of mTOR signaling. Through targeted sequence deletion studies, two regions within *SNHG6*, loop 1 and loop 3, have been identified that specifically interact with FAF2 and mTOR, respectively. These interactions enhance FAF2-mTOR binding and the activation of mTOR signaling at the ER-lysosome contact sites and subsequently cholesterol biosynthesis (Fig. 3c). Of note, *SNHG6* inactivation blocks mTOR signaling and inhibits tumor growth in a patient-derived hepatoma xenograft model. Hence, *SNHG6* acts as a tether that is important for lysosomal recruitment and activation of mTOR by enhancing the ER-lysosome contacts. These findings suggest a new role for lncRNAs in organelle communication that facilitates and modulates organelle-specific crosstalk and signaling for example in the sensing of nutrients or other biomolecules, such as cholesterol, to maintain cellular and organismal homeostasis.

## lncRNAs in stress-related liquid–liquid phase separation

The biophysical process of liquid–liquid phase separation (LLPS) is nucleated by multivalent interactions of RNAs and RNA binding proteins that often contain modular domains or intrinsically disordered regions (IDRs). LLPS leads to the formation of membrane-less compartments comprised of phase-separated RNA-protein condensates throughout the cell that have been described in many cell and tissue types and implicated in various biological functions (Banani et al. 2017). Phase-separated granules can for example control the specificity and the kinetics of biochemical reactions, sequester biomolecules to limit their available concentrations, or dynamically modulate complex biological processes through the active regulation of phase-separated condensates. lncRNAs have been increasingly shown to participate in LLPS and in stress-related responses involving phase separation and phase-separated compartments (Onoguchi-Mizutani and Akimitsu 2022).

**Fig. 4:**
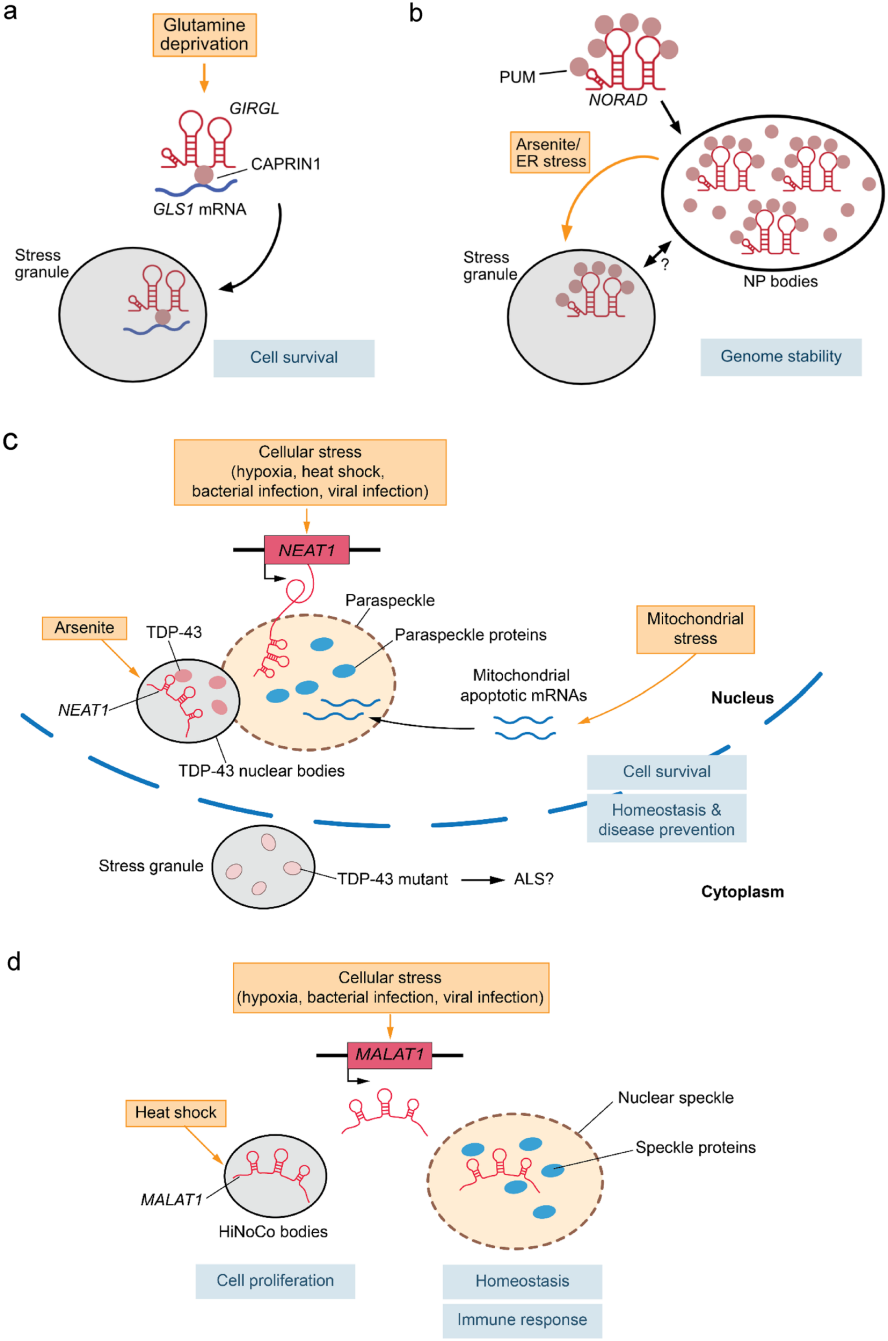
lncRNAs in stress-related liquid–liquid phase separation. **a** Induction of the lncRNA *GIRGL* upon glutamine deprivation and subsequent recruitment of *GLS1* mRNA to stress granules in a lncRNA containing ribonucleoprotein complex. **b** *NORAD* binding to PUMILIO proteins (PUM) and the formation of *NORAD*-PUMILIO (NP) bodies as well as potential, yet to be shown, crosstalk with other condensates, such as stress granules. **c** Increased *NEAT1* expression and paraspeckle formation upon various types of stress and the role of *NEAT1*-dependent TDP-43 nuclear bodies. (ALS = amyotrophic lateral sclerosis). **d** Induction of *MALAT1* upon various types of stress and the role of *MALAT1* in the heat-induced noncoding RNA containing nuclear bodies (HiNoCo bodies)

Different types of cellular stress, including ER stress, oxidative stress, heat shock and hyperosmotic stress, result in the formation of cytoplasmic ribonucleoprotein granules, called stress granules. While the protein composition, including proteins like the G3BP stress granule assembly factor, TIAR, TIA1, or the eukaryotic translation initiation factor 4E (eIF4E), has been well-documented, relatively little has been known about the RNA composition of these phase-separated stress condensates (Campos-Melo et al. 2021). Studies on the stress granule transcriptome have revealed that mainly transcripts with poor translation and relatively long sequence length, including mRNAs as well as noncoding RNAs, are recruited and enriched in these ribonucleoprotein condensates (Khong et al. 2017; Namkoong et al. 2018). While stress granule formation does not per se appear to be a stress-specific response, it has been shown that certain stress types such as ER stress, heat shock or arsenite treatment can recruit similar, yet distinct subsets of RNAs to stress granules dependent on their translational activity, their length and the presence of AU-rich elements. However, how specific RNAs or mRNAs may be recruited to stress granules and translationally inhibited in response to a certain stress has remained an open question. In a recent study (Wang et al. 2021), a novel stress-related lncRNA was shown to recruit a specific mRNA within a ribonucleoprotein complex to stress granules. This less than one kb long noncoding RNA was identified in a screen for glutamine starvation-responsive lncRNAs and was hence termed *glutamine insufficiency regulator of glutaminase lncRNA* (*GIRGL*). Glutamine is an energy source as well as a source of nitrogen for the synthesis of many biomolecules. As a first step of glutamine utilization, glutamine is converted into glutamate by an enzyme called glutaminase, which is encoded by the genes *GLS1* and *GLS2*. Glutamine deprivation results in downregulation of glutaminase (*GLS1*), which has been otherwise shown to be overexpressed in human cancers and to promote tumor growth. Glutamine starvation leads to *GIRGL* upregulation and inhibition of glutamine metabolism via translational repression of *GLS1*. *GIRGL* interacts with the stress granule-associated RNA binding protein CAPRIN1 (cell cycle associated protein 1) to promote the formation of a *GIRGL*-CAPRIN1-*GLS1* ribonucleoprotein complex that is targeted to stress granules, preventing *GLS1* translation (Fig. 4a). Under normal glutamine supply, *GIRGL* suppresses tumor cell growth in colon cancer cells, whereas under glutamine starvation *GIRGL* facilitates cell survival under prolonged glutamine deprivation, which may help tumors to adapt to a glutamine-low microenvironment. This suggests a role for a lncRNA in the recruitment of specific stress-related mRNAs to stress granules, where they are translationally repressed.

Another noncoding RNA that is associated with stress granules is the *noncoding RNA activated by DNA damage* (*NORAD*) (Khong et al. 2017; Namkoong et al. 2018). *NORAD* and its interacting RNA binding proteins PUMILIO1 (PUM1) and PUM2 are strongly enriched in stress granules upon arsenite treatment and ER stress, although neither *NORAD* nor PUMILIO are required for each other’s recruitment to stress granules under ER stress. This suggests that *NORAD* is targeted to stress granules via other RNA binding proteins, such as TIAR or TIA1, presumably through AU-rich elements within the *NORAD* sequence, which is in line with the general notion that RNA targeting to stress granules requires multiple RNA-protein, protein-protein, and RNA-RNA interactions (Matheny et al. 2021). Interestingly, *NORAD* has been demonstrated to nucleate another phase-separated cytoplasmic condensate that is distinct from stress granules and that has been termed *NORAD*-PUMILIO (NP) body, which is present at steady state, induced upon DNA damage and critical for maintaining genome stability (Elguindy and Mendell 2021). *NORAD* has been initially described as a 5 kb long, predominantly cytoplasmic noncoding RNA that is induced by DNA damage in a p53-dependent manner and that regulates the activity of PUMILIO RNA binding proteins (PUM1 and PUM2) (Lee et al. 2016; Tichon et al. 2016). PUMILIO proteins bind to a very specific sequence, also referred to as PUMILIO response elements or PREs, in predominantly the 3’ untranslated regions (UTRs) of mRNAs, negatively affecting their stability and translation (Goldstrohm et al. 2018). *NORAD* harbors at least 15 PREs and represents the preferred RNA binding partner of PUM2 in human cells. Loss of *NORAD* results in PUMILIO hyperactivity and the repression of PUMILIO target mRNAs, which leads to chromosomal instability and mitochondrial dysfunction in human and mouse cells as well as to degenerative phenotypes in mice, resembling premature aging (Elguindy et al. 2019; Kopp et al. 2019; Lee et al. 2016; Tichon et al. 2016). Although *NORAD* is abundantly expressed and represents a multivalent binding platform with multiple PREs per molecule, it has not been understood how *NORAD* can compete with the transcriptome-wide pool of PRE-containing RNA targets through a simple titration model of PUMILIO proteins. Elguindy and Mendell recently showed that *NORAD* facilitates LLPS which enables the sequestration of a super-stoichiometric number of PUMILIO proteins into NP bodies, thereby out-competing the transcriptome-wide pool of PRE-containing RNA targets (Elguindy and Mendell 2021). They further demonstrated that this phase separation event is critical for *NORAD*’s molecular function, which is to regulate PUMILIO activity and maintain chromosomal stability (Fig. 4b). Interestingly, it has been shown that these *NORAD* condensates appear to increase in size when cells undergo DNA damaging stress (Elguindy et al. 2019; Elguindy and Mendell 2021), suggesting that NP bodies may be actively regulated upon different types of cellular stress in order to fine-tune PUMILIO activity depending on the cellular demands. Future investigations will be required to fully characterize and understand the role of the *NORAD*-PUMILIO axis and the formation of NP bodies and potential cross-talk with other condensates in the cellular stress response.

While stress granules and NP bodies are two examples of phase-separated condensates in the cytoplasm, there are also several ribonucleoprotein granules in the nucleus, such as nuclear stress bodies, nuclear speckles and paraspeckles, which have been also implicated in cellular stress response (Onoguchi-Mizutani and Akimitsu 2022). A well-characterized example of these nuclear condensates are the *NEAT1*-induced paraspeckles, which are thought to be involved in the regulation of transcription, RNA editing and nuclear retention of selected mRNAs (Fox and Lamond 2010). *NEAT1* is a nucleus-retained lncRNA that associates with paraspeckles and that exists in two isoforms, a shorter 3.7 kb long isoform called *NEAT1_1* (or *MENε*) and a longer ~ 23 kb long isoform called *NEAT1_2* (or *MENβ*) (Clemson et al. 2009; Sasaki et al. 2009; Sunwoo et al. 2009). The longer isoform *NEAT1_2* has been shown to be critical for paraspeckle formation (Naganuma et al. 2012; Yamazaki et al. 2018). *NEAT1_2* is characterized by a modular structure, in which the middle module binds to the RNA binding protein non-POU domain containing octamer binding (NONO), which is necessary and sufficient for the initiation of LLPS and paraspeckle formation (Yamazaki et al. 2018). Interestingly, paraspeckle formation is highly dynamic and can be regulated by various types of stress. It has been shown that *NEAT1* and paraspeckles are involved in the response to viral and bacterial infection (Imamura et al. 2014, 2018). Furthermore, *NEAT1* and paraspeckles have been reported to be induced by stress conditions like heat shock and hypoxia (Choudhry et al. 2015; Godet et al. 2022; Lellahi et al. 2018). *NEAT1* is also induced upon activation of the integrated stress response following ER stress in multiple myeloma cells, where it interacts with the apoptosis antagonizing transcription factor (AATF) in paraspeckles to prevent R-loop accumulation and activation of an inflammatory response (Bruno et al. 2022). In addition, mitochondrial stress or depletion of mitochondrial genes can also result in an induction of *NEAT1* and increased numbers of paraspeckles, which leads to nuclear retention of mitochondrial and apoptotic mRNAs and subsequently to improved cell survival (Wang et al. 2018). Recently, it has been shown that *NEAT1* promotes LLPS and the formation of TAR DNA binding protein 43 (TDP-43) nuclear bodies (Wang et al. 2020). Upon arsenite stress, TDP-43 is targeted partly to stress granules in the cytoplasm and mostly to *NEAT1*-mediated nuclear bodies that overlap with paraspeckles. TDP-43 has been associated with the development of amyotrophic lateral sclerosis (ALS) and an ALS-related mutation (D169G) of TDP-43 has been shown to impair *NEAT1*-mediated LLPS and nuclear body formation, resulting in an accumulation of TDP-43 in stress granules instead (Fig. 4c). Importantly, nuclear TDP-43 appears to be cytoprotective, whereas cytoplasmic TDP-43 is thought to be involved in the development of ALS. These findings suggest a critical function of *NEAT1* and paraspeckles in response to a broad range of cellular stress types. The dynamic regulation of LLPS by varying the concentrations of a lncRNA may provide a powerful tool to the cell to adapt to different stress conditions, to titrate the necessary stress response, and to prevent pathophysiological conditions, such as ALS.

A stress-related function has been also ascribed to the *metastasis-associated lung adenocarcinoma transcript 1* (*MALAT1*). Initially, *MALAT1* has been associated with lung tumor progression and metastasis (Ji et al. 2003). Later studies showed that *MALAT1* localizes to nuclear speckles (Hutchinson et al. 2007; Wilusz et al. 2008), a ribonucleoprotein granule that is known to contain RNA splicing factors and transcriptional regulators (Spector and Lamond 2011). Interestingly, *MALAT1* is dispensable for the formation of these nuclear compartments, and neither do *Malat1*-deficient mice display abnormalities in pre-mRNA splicing nor do they have obvious developmental defects or a measurable reduction in viability (Eissmann et al. 2012; Nakagawa et al. 2012; Zhang et al. 2012). These findings raised the question regarding the actual biological function and the physiological role of *MALAT1*. Since then, *MALAT1* has been shown to be induced upon different types of cellular stress, such as hypoxia in endothelial cells (Michalik et al. 2014), treatment with chemotherapeutic drugs in multiple myeloma (Handa et al. 2017), or lipopolysaccharide treatment in macrophages (Zhao et al. 2016). Recently, *Malat1* has been also shown to be involved in CD8^+^ T cell differentiation upon viral infection (Kanbar et al. 2022), suggesting an important role in the immune response. Interestingly, upon heat shock *MALAT1* appears to redistribute from nuclear speckles to a distinct nuclear compartment named heat-inducible noncoding RNA containing nuclear bodies (HiNoCo bodies) (Onoguchi-Mizutani et al. 2021). Although the precise composition of the HiNoCo bodies and the mechanism of how these granules are formed are currently unknown, these lncRNA containing nuclear granules may function as critical biosensors in the response to sudden temperature changes (Fig. 4d). While more research is needed to fully understand the role of noncoding RNAs like *MALAT1* in the mammalian stress response, these examples demonstrate how lncRNAs can potently modulate the output of different stress responses through the efficient and timely regulation of cellular phase-separated condensates.

## Conclusion

We have categorized the molecular functions of stress-related lncRNAs into three general classes: (I) gene regulatory functions in *cis* or *trans*, (II) roles as molecular scaffolds or tethers, and (III) functions in the regulation and formation of phase-separated condensates (Table 1). These three general modes of action and the underlying molecular mechanisms presented in this review should be however regarded as an initial framework that will need continuous revision. As new biochemical, molecular and genetic technologies evolve, an increasing number of stress-related lncRNAs and new molecular modes of action will be identified. On the other hand, individual functions of the currently existing and known lncRNAs may need to be revised or complemented by novel models based on future findings.

**Table 1:** Summary of stress-related lncRNAs and their modes of action

lncRNA	Stress response	Molecular mechanism
Gene regulatory functions in *cis* or *trans*		
* PINCR*	DNA damage	Gene regulation in *trans* via matrin-3 and p53
* PANDAR*	DNA damage	Gene regulation in *trans* via sequestration of NF-YA
* lncRNA-p21*	DNA damage	Regulation of *CDKN1A* expression in *cis*
* PVT1*	DNA damage and oncogenic stress	Regulation of *MYC* expression in *cis*
Roles as molecular scaffolds or tethers		
* DINO*	DNA damage	Interaction with and stabilization of the p53 protein
* PURPL*	DNA damage	Destabilization of the p53 protein via interaction with MYBBP1A
* SPARCLE*	DNA damage	Cofactor for caspase 3-mediated PARP1 cleavage
* GUARDIN*	DNA damage and oncogenic stress	Decoy for *miR-23a* and stabilization of BRCA1 via BARD1
* GAS5*	Nutrient deprivation	Disruption of a TCA cycle enzyme complex and decoy for glucocorticoid receptors
* NKILA*	Immune activation and hypoxia/ischemia	Inhibition of IκB phosphorylation and NF-κB signaling
* SNHG6*	Metabolism/cholesterol homeostasis	Lysosomal recruitment of mTOR and activation of mTOR signaling
Functions in phase-separated condensates		
* GIRGL*	Glutamine deprivation	Recruitment of *GLS1* mRNA to stress granules via CAPRIN1
* NORAD*	Various stress types	Formation of NP bodies to restrain PUMILIO activity
* NEAT1*	Various stress types	Formation of paraspeckles and another stress-related nuclear condensate (TDP-43 nuclear bodies)
* MALAT1*	Various stress types	Component of nuclear speckles and another stress-related nuclear condensate (HiNoCo bodies)

Regardless, lncRNAs are perfectly suited molecules to exert important functions in the response to various types of cellular stress. Since there is no need for an additional step to translate the RNA, as it is the case for protein effectors and their encoding mRNAs, lncRNAs can be almost instantly induced upon the emergence of a stress condition, once the stress signal has been recognized and processed (e.g. in the cases of *PANDAR* and *DINO*). The functionality of a stress-induced lncRNA can be further adjusted through its abundance and/or stability, which can induce effects that are rather locally and timely restricted (e.g. in the case of *Pvt1b*) or extended to distant genomic or cellular sites (e.g. in the case of *PINCR* or *NORAD*). And lastly, the often-modular structure of lncRNAs make them pleiotropic effectors that can initiate a multifaceted stress response depending on the respective cellular demands and experienced stress conditions (e.g. in the case of *GAS5*). The modular structures of lncRNAs and how they can confer functionality to the noncoding transcript will be an interesting area of future research as well as the remaining issue of the frequently seen imbalance between the abundance of the lncRNA and the abundance of the interacting RNA or protein binding partners. While a few lncRNAs may simply display a sufficient abundance to support their ascribed molecular mode of action, others may fail to do so. However, a lncRNA’s molecular characteristics and underlying mode of action, such as the formation of phase-separated condensates or the recycling of low abundant lncRNA molecules in a certain cellular reaction (e.g. in the case of *SPARCLE*), may provide plausible evidence for its mode of action, even if the transcript is less abundant and expressed at a sub-stoichiometric ratio (Unfried and Ulitsky 2022). Precise biochemical testing of RNA-protein and RNA-RNA interactions, deciphering the RNA structure and associated structure-function relationships, as well as rigorous genetic analysis of the underlying modes of action will help to identify novel lncRNAs with valid molecular functions not only in the mammalian stress response, but also in normal cellular homeostasis, physiology and disease.

## Data Availability

Not applicable.
